# The Odyssey from Symptom to Diagnosis of Pulmonary Hypertension from the Patients and Spouses Perspective

**DOI:** 10.1177/21501327211029241

**Published:** 2021-07-03

**Authors:** Bodil Ivarsson, Anders Johansson, Barbro Kjellström

**Affiliations:** 1 Lund University and Medical Services University Trust, Region Skåne, Lund, Sweden; 2 Karolinska Institutet, Stockholm, Sweden; 3 Lund University and Skåne University Hospital, Lund, Sweden

**Keywords:** diagnostic errors, help-seeking behavior, partner communication, rare disease, sign and symptom

## Abstract

**Introduction/objective:**

Diagnostic delays in pulmonary arterial hypertension (PAH) and chronic thromboembolic pulmonary hypertension (CTEPH) are related to increased morbidity and mortality. The risk of a delayed, or even a missed, diagnosis is high as the conditions are rare. The aim was to describe patients’ and spouses’ experiences of the journey from the first symptom to an established diagnosis.

**Methods:**

A secondary analysis of 31 transcripts, based on 2 primary datasets containing interviews with 17 patients and 14 spouses, was carried out and analyzed according to qualitative content analysis.

**Results:**

One overarching category was revealed from the content analysis; *“The journey from doubt and hope to receive the diagnosis.*” Five subcategories were identified as: *overall experiences; ignoring symptoms; seeking primary care/hospital specialty care; blame and stigma; and finding a pulmonary hypertension specialist clinic.* The main finding was that both patients and spouses experienced that waiting for a diagnosis and the deteriorating state of health led to anxiety and frustration. The knowledge about rare diseases among health professionals needs to be improved to enable a timelier diagnosis and initiation of treatment.

**Conclusion:**

Patients’ and spouses’ lives were negatively affected by having to search for a correct diagnosis. In order for health care to identify rare diseases earlier, a well-functioning and responsive health care system, in primary care as well as in specialist care, is needed. Symptoms like breathlessness and fatigue are often unspecific but should not be ignored. Keeping the patient and spouse in the loop, and providing information that the search for an answer might take time is essential for health care providers to create trust.

## Background

The journey from the first symptom to diagnosis can be frustrating for the patient and this is especially true for patients with pulmonary arterial hypertension (PAH) and chronic thromboembolic pulmonary hypertension (CTEPH).^1^ These are rare and life-threatening diseases that present with unspecific symptoms like fatigue and shortness of breath.^2-4^ The symptoms are often mistaken for other common and less serious illnesses such as asthma, flue, or stress related diseases.^5^

With the availability of disease-specific treatments, the overall survival of patients with PAH and CTEPH have improved.^6^ But a delay in the start of treatment may have grave consequences for the prognosis.^7^ The intention is to detect and diagnose PAH and CTEPH at an early stage, so that the progressive deterioration of the pulmonary arteries can be stopped.

Patients with PAH or CTEPH have described the time to diagnose as a long journey including visits to multiple physicians and institutions.^1,8^ For many patients, this journey will take more than a year^1,2,8-10^ and they may endure feelings of anxiety, fear, and self-doubt before a correct diagnosis is established.^8^ In addition, it is not uncommon that more than a year elapse from the first symptom until seeking care.^1,9^ Patients diagnosed at an age younger than 35 years, said they waited even longer.^1^ With the long delay from first symptom to a diagnose, symptoms will escalate and affect the quality of life negatively.^11^ This emotional uncertainty may also affect the family.^5,12^ The patients and their families experiences and perspective of the time before a PAH or CTEPH diagnosis is still relatively unexplored.

The aim of the present interview-based study was to describe the patients, as well as the spouses, experience of a journey from the first symptom to an established diagnosis.

## Methods

### Design

A secondary analysis of existing qualitative data (transcripts of interviews) from 2 previously published studies was conducted.^13,14^ Secondary analysis of qualitative data use already existing qualitative data with an aim to bring to find answers to new research questions, different from those asked in the original research.^15^

## Ethics Procedures

The Regional Research Ethics Committee in Lund, Sweden approved the studies (LU 2011/364). Informed consent was obtained from all participants prior to conducting the interviews.

## Selection of Informants

Transcripts of interviews from 2 studies were used in this secondary analysis.^13,14^ The first study included interviews with 17 patients with PAH or CTEPH and the second analysis included 14 spouses to patients with PAH or CTEPH. Nine of the participating patients and spouses were a couple. The one-to-one interviews were conducted by one of the researchers (BI) and took place in person or over the telephone and lasted between 13 and 67 min. A verbatim transcription of audio-recorded interviews was made later. Demographic data of the study population are shown in Table 1. Further details about the original study design and methodology used for analyses can be found in the earlier publications.^13,14^

**Table 1.** Demographic Data and Disease Characteristics of the Patients (n = 17) and Spouses (n = 14).

	Patients	Spouses
Sex (women/men)	13/4	5/9
Age (years)	60 (28-73)	68 (40-87)
Time since diagnosis (years)	4 (1-12)	
Education		
Elementary school (n)	4	1
High school (n)	6	8
College/university (n)	7	5
Current occupation		
Full time job (n)	3	7
Part time job (n)	5	
Disability/retirement pension	9	7

Data are shown as median (min-max) or numbers.

### Data Analysis

Transcripts from interviews were analyzed using Microsoft Word^®^ 2016 Tools^16^ by means of manifest qualitative content analysis inspired by Graneheim and Lundman.^17^ Through a process of reading and re-reading the new research question, 1 author (BI) identified a new area. Significant meaning units were identified, condensed, and coded. The codes were then convened in sub-categories and abstracted into 1 main category. The other authors (AS and BK) independently and critically examined the sub-categories and the main category and reflected on them. In a discussion between the 3 authors and after ensuring accuracy in all steps, the final analysis was performed to confirm the content and reach a consensus as to strengthen the credibility of the results.^17^ To improve the validity of the categories and to illustrate the patients’ and spouses’ experiences, original direct quotations are presented in the text. In the quotations presented, each patient was allocated a code number prefixed by “P,” and the spouse was given a code number prefixed by “S.”

## Results

**Figure 1.**
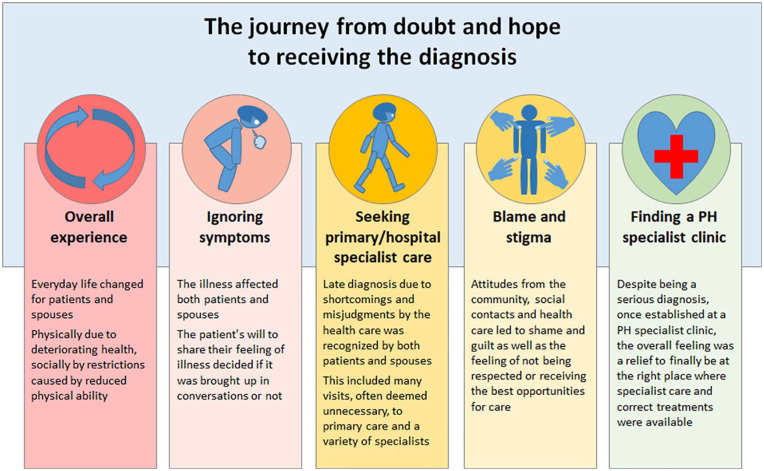
Infographic model with main category and sub-categories.

The analysis revealed 1 main category: “The journey from doubt and hope to receiving the diagnosis” and 5 subcategories (Figure 1).

## Subcategory 1: Overall Experience

Everyday life changed both for patients and for spouses. Physically due to the deteriorating health as well as socially because of the constraints the reduced physical ability caused.

### Patients

Most patients had a feeling of malaise before the diagnose, this period ranging for a couple of months and up to as long as 4 years. These feelings included uncharacteristic fatigue and not being able to participate in normal activities like walking stairs or uphill, cycling, dancing, playing golf, or inviting guests to their home. This would go on, sometimes for quite a while, without them seeking medical care for their unease. Severe palpitations, shortness of breath, and anxiety were common symptoms and some patients had experienced dizziness and in some instances they had even fainted.

*“I could not do what I usually do, instead I had to pause and finally, I just couldn’t do anything anymore. Finally, I had to rest three times just to climb one flight of stairs to get to the upper floor.”(P 2)*

### Spouses

The spouses recalled their own sense that their partner showed deviances in their behavior and that they [spouses] perceived this as symptoms of fatigue and decreased physical strength. For most partners, the symptoms had slowly sneaked into their lives, but for others, it had started quite abruptly. The spouses generally kept the feeling of unease to themselves.

*“In the beginning, I did not really take it seriously. Instead, I just took it with a grain of salt.” (S 4)*

## Subcategory 2: Ignoring Symptoms

The illness affected both the patients and the spouses but it was the patient’s willingness to share their feeling of illness that decided if it was brought up in conversations or not.

### Patients

Some patients did not want to acknowledge their symptoms and delayed, consciously, or unconsciously, the contact with the health care system. In some cases, they also hid their feeling of illness from the partner.

*“I felt out of breath and had a lot of anxiety. . . it felt like I had something stuck between the rib cage and the backbone. But I did not go to the doctor—of course—instead, I got pregnant.” (P 17)*

### Spouses

Some spouses were convinced that the partner took an ostrich approach—if you cannot see it, it does not exist. Thus, the spouses did not really sense the partners’ perception of illness and were not able to support them in seeking medical care.

*“I think I kept me in the blue, we did have a newborn. . . she [the patient] would not have told me how she felt anyway, she says she would not have coped with having me worry about it.” (S 3)*

## Subcategory 3: Seeking Primary Care/Hospital Specialty Care

Late diagnosis due to shortcomings and misjudgements by the health care they had sought was acknowledged among both the patients and the spouses. This included many visits, often deemed unnecessary, to primary care and a variety of specialists.

### Patients

Once the patients had made contact with the health care system, they were commonly misdiagnosed as pneumonia, COPD, asthma, alcohol abuse, or panic disorder. Others experienced that their existing conditions, like thyroid disease, menopause, or overweight were used to explain their symptoms. Most patients were repeatedly bounced between primary care and various specialists. Some patients had even been declared healthy both in primary as well as specialist care.

*“I sought help in the primary care, they tested my metabolism and I got treatment for thyroid disease. I complained about shortness of breath, but the doctor just increased the dose of the medicine. But finally, I went to the clinic twice the same week. When I called they said ‘you were just here this Monday’, and then I said, yes, but I cannot live like this any longer, I cannot breathe.” (P 16)*

### Spouses

Some spouses had taken note of their partner’s countless visits in health care before receiving a diagnosis, but kept themselves at a distance from it and reconed that they should have been a greater support. Other spouses had been partly involved in the partners “pursuit of healing and improvement” or had actively tried to help their partners to receive adequate care.

*“I was with him at every visit to the primary care clinic. He got asthma inhalers, one after the other. After a year I told them [the primary care doctor], you need to do something about this and that’s when they sent him to the hospital who then, in their turn, referred him to the PAH-centre.” (S12)*

## Subcategory 4: Blame and Stigma

Attitudes from the community, social and health care led to shame and guilt as well as the feeling of not being respected and not receiving the best opportunities for care.

### Patients

Due to ignorance and prejudice among health care professionals, the surrounding society, and not the least, by themselves, patients could feel shame and stigma regarding physical strength, body weight, smoking, and their anxiety. This led to them accepting their own and the surroundings prejudices and opinions, which led to delays in seeking help and contact with the health care system for their symptoms.

*“It had been a full year and I had felt really miserable before being diagnosed. But what I really reacted on was the attitude at the primary care center. I was in a bad state already when I sought care there a year before and all the physician said was that I had some overweight and should lose weight.”(P 14)*

### Spouses

Some spouses realized, at a later stage when there was a diagnose, that they had ignored their partner’s illnesses due to inadvertency and unawareness.

*“My wife had felt a little tired, she had to pause and take some deep breaths and did not have any energy. My response might not have been the most adequate, so we bought a treadmill, an exercise bike and other things like that because I felt she had to exercise more. There are so many pieces in the puzzle that have now fallen into place and explain the full picture of her disease. It is just that I have been so incomprehensible.” (S 2)*

## Subcategory 5: Finding a Pulmonary Hypertension Specialist Clinic

Despite the serious diagnosis being established at a PH specialist clinic, the overall feeling was a reassurance to finally have landed at the right place in the health care system where specialist care and correct treatments were available.

### Patients

Most patients’ perception was that it was just a coincidence that finally got them referred to a PH specialist clinic and the correct diagnosis. It was often stated that the reason was that their primary physician was not available and instead they met either a medical student that knew about PH or a new physician that had just read an article about PH. Patients with symptoms so severe they had to seek care at an emergency department often underwent a series of examinations that raised suspicion of the diagnosis and earned them a quick referral to a PH specialist clinic. Despite the serious nature of the disease, everyone expressed relief to have received a correct diagnosis.

*“I had been to many health care visits before I came to the right place. In the primary care clinic, there was a medical student who was doing his residency. My primary doctor wanted him to check me out first. My view on the situation was that there was no reason to say anything about my problems because he will not know, it is just a young guy. But he knew everything, it was incredible, he even knew that the PAH-team existed.” (P 1)*

### Spouses

The spouses also described a feeling of relief that their partners had finally got a conclusive diagnosis and by that the possibility for a treatment that would help. Several spouses described a sense of powerlessness to not have been able to affect their partner’s possibility to get help and thereby an earlier diagnosis and treatment.

*“When we had an urgent referral, we came to the right persons, and then it was just a paved road forward. . . .and then, at the PH-center, we have the physician of the world. But finding the way to get there was not easy.” (S 3)*

## Discussion

This study contributes to the understanding of how patients, now diagnosed with PAH and CTEPH, experienced the time from the first signs of illness to the diagnosis of chronic life-threatening disease. It also brings understanding to the spouses’ perspective of the same period. Most patients had experienced an insidious feeling of illness and unspecific symptoms for quite a long time before seeking medical attention. Reasons for not seeking care, despite symptoms, and health problems, have been recognised as a multifaceted mix of physical, psychological, and social factors.^18^ These factors relate to the perception of symptom severity that includes feelings of anxiety or ignorance intertwined with the feeling of optimism. Other factors are the availability and previous experiences of the health care system as well as the patients age.^18^ The present study suggests that delays from the first symptom to correct diagnosis can be partly related to the patient’s own denial of feeling ill and thus, more difficult to alter. However, the study also identified delays related to health care which leaves room for attention and possible improvements. Issues that influence the decision to pursue and select health care as well as the continuity of health care warrant attention. This highlights the need for diagnostic tools that combine symptoms, common as well as uncommon, to suggest possible diagnoses.^19^ For patients with PAH or CTEPH, systematic evaluation of shortness of breath would be a good start.^20^ With the help of leading questions suggested by the tool, rare diseases might be able to detect as well as dismissed. This is of extra relevance for the primary care, taking in account that with the low incidence of patients with PAH or CTEPH, estimated to 2 to 10 patients per million inhabitants,^4^ most physicians in primary care will not meet one of these patients in their clinical practice.

Patients in the present study said they tried to not display their symptoms while spouses described having a sense that their partners were hiding something. This shows the difficulty with social interaction even in a family constellation. It shows the importance of social support where the response from family and friends to health problems will affect the patient’s journey from the first symptom to a diagnosis.^18,21^ While it is difficult for health care to influence the family’s interpersonal relations, the importance of health care staff and facilities being readily available and welcoming is undisputable.

When patients acknowledge their symptoms and seek help, they have reached a point when they look for an answer, a relief, and a cure. This study revealed that both patients and spouses expected the primary health care physicians to possess enough medical knowledge to be able to address their symptoms and refer them to the right specialist. When these expectations were unmet, patients described that they felt misunderstood and dissatisfied while the spouses more often described a feeling of frustration. Primary care and emergency departments meet a lot of patients with unspecific symptoms that cannot be directly traced to a specific diagnosis.^22,23^ Thus, the generalists in primary care are the ones who should dare to think outside the box and look for patterns that might lead to a rare disease.^24^ For example, when symptoms like shortness of breath and fatigue cannot be explained as asthma, COPD or panic disorder, readily available tests like Nt-proBNP by a blood sample and ventricular function by a bedside echo can be a first, and large, step toward a correct diagnosis.^25^ With this, unnecessary visits, which patients and spouses in this study described as having occurred, might have been avoided. When the Swedish patient association did a member survey and asked about the time before the diagnosis, a quarter of the patients said they had been to 10 or more visits to a primary care health facility before being referred to a PH specialist clinic.^1^ A British PH study showed similar results with a long and bumpy road in the health care system before being referred to the PH specialist clinic.^9^ Whether it is a risk or a possibility that patients and spouses search for a diagnosis on the internet should be left unsaid. Although patients and spouses in this study did not bring up if they had searched on the internet prior to diagnosis, it is today common to first self-diagnose with the help of the internet and then seek help, hoping to get the diagnosis confirmed by a physician.^26^ Social media is today used by many and those with undiagnosed conditions are no exception. There is even a tool being developed where artificial intelligence together with social media will connect people with similar stories about their diagnostic odyssey, handling of symptoms, and possible diagnoses.^27^ This was based on the development of a questionnaire where patients with rare diseases were shown to share pre-diagnosis experiences and that the patterns that evolved might be useful to direct the patient toward the correct diagnosis.^19^

Both the patients and the spouses expressed a wish to have been listened to and a greater extent of trust by the health care staff they met during their journey to a diagnose. Person-centred care, that is, the partnership between patients and health care professionals, aims to improve communication and contact between patients, families, and caregivers.^28^ The key in person-centred care, and this cannot be emphasised enough, is to listen and interpret the patient’s verbal and non-verbal expressions that, together with other examinations, form the basis for planning individualized care.^28^

The present study showed that patients and spouses appreciated being seen at a PH specialist clinic, whose purpose is to provides good holistic care including treatment, support, and accurate information about the disease in a multi-professional environment.^4,29^ Despite the changes and challenges a chronic disease infer on life, patients and spouses adapt and it might even strengthen a relationship.^30^ The present study supports this by showing that even with a grim diagnosis as PAH or CTEPH, patients, and the spouses shared a feeling of relief that the search was over.

The main implication of this study is to help the health care professionals who meet the patients on their way to diagnosis, to better understand this journey and develop pedagogical strategies. For many of these patients, when they first seek help for their symptoms, it is in the primary care. There is a need to convey an understanding of the patients’ condition and to explain to the patient, the detective work that is sometimes needed to find a correct diagnosis and that will cause time delay. It is important that the patients know they are not being ignored or dismissed. To maintain a balance between providing adequate information and support and ensuring the patients’ autonomy, the involvement of the family is essential.

### Limitations

Secondary analysis is performed to not waste rich data.^15^ In the present study the patients and spouses had taken the time to be interviewed and deliver a rich base of information. Although 10 years and more could have passed since diagnosis, patients and spouses vividly recalled the time before diagnosis. While people who have experienced emotional events will remember them even if a long time has passed since they occurred,^31^ it cannot be completely ruled out that recall bias might have affected statements in the present study. The uneven number was because not all patients had a spouse or that the spouse of a participating patient did not want to take part. In other instances, the patient did not want to participate, but the spouse accepted.

## Conclusion

The lives of the patients and spouses were negatively affected by having to search for a correct diagnosis. In order for the health care to identify rare diseases earlier, a well-functioning and responsive health care system, in primary care as well as in specialist care, is needed. Symptoms like breathlessness and fatigue are often unspecific but should not be ignored. Keeping the patient and spouse in the loop, and providing information that the search for an answer might take time is essential for health care to create trust.
